# Efficacy, safety, and in-hospital outcomes of subcutaneous versus transvenous implantable defibrillator therapy: A meta-analysis and systematic review

**DOI:** 10.1097/MD.0000000000015490

**Published:** 2019-05-13

**Authors:** Chao-Feng Chen, Chao-Lun Jin, Mei-Jun Liu, Yi-Zhou Xu

**Affiliations:** a Department of Cardiology, Affiliated Hangzhou First People's Hospital, Zhejiang University School of Medicine; b Nanjing Medical University, Hangzhou, Zhejiang, China.

**Keywords:** clinical outcomes, subcutaneous implantable cardioverter-defibrillators, sudden cardiac death, trans-venous implantable cardioverter-defibrillators

## Abstract

**Background:**

Lead-related complication is an important drawback of trans-venous implantable cardioverter-defibrillators (TV-ICD). The subcutaneous ICD (S-ICD) was developed to overcome ICD lead associated complications; however, whether the S-ICD confers enhanced clinical benefits compared with TV-ICD remains unclear. The present systematic review and meta-analysis aimed to assess TV-ICD and S-ICD for safety, efficacy, and in-hospital outcomes in the prevention of sudden cardiac death (SCD) in patients not requiring pacing.

**Methods:**

The Medline, PubMed, EmBase, and Cochrane Library databases were searched for studies comparing TV-ICD and S-ICD.

**Results:**

A total of 9 eligible studies, including 5 propensity-matched case–control, 3 retrospective, and 1 cross-sectional studies were identified, assessing 7361 patients in all. Pool analyses demonstrated that SICD were associated with lower lead-related complication rates [odds ratio (OR) = 0.13; 95% confidence interval [CI] 0.05–0.33; *I*^2^ = 0%], and S-ICD was more beneficial in terms of reducing ICD shocks [OR = 0.48; 95% CI 0.32–0.72, *I*^2^ = 4%]. In addition, the patients administered S-ICD tend to have shorter length of hospital stay after implantation (SMD = −0.06; 95% CI −0.11 to 0.00, *I*^2^ = 0%) and reduce total complication rates (OR = 0.72; 95% CI 0.50–1.03; *I*^2^ = 18%), non-decreased quality of life (QoL). Moreover, both devices appeared to perform equally well with respect to infection rate and death.

**Conclusions:**

Available overall data suggested that S-ICD is associated with reducing lead-related complications, ICD shocks. In addition, S-ICD has tendency to shorten hospitalization and reduce total complications, although the difference is no significant. Equivalent death rate, infection, and QoL were found between 2 groups. Therefore, S-ICD could be considered an alternative approach to TV-ICD in appropriate patients for SCD prevention.

## 1 Introduction

Cardiovascular disease has become the foremost cause of death in many countries.^[1]^ Despite advances in cardiovascular care, sudden cardiac death (SCD) still accounts for half of all cardiovascular deaths.^[2]^ Most of randomized trials demonstrated that the implantable cardioverter defibrillator (ICD) could improve survival in patients at risk of SCD, and, this device has emerged as the established therapy for SCD from ventricular tachyarrhythmia, both in primary and secondary prevention strategies.^[3]^ Nevertheless, the conventional ICD system is associated with significant complications, which remain an important drawback, both perioperative and during follow-up.^[4]^ Trans-venous lead is the source of most mechanical adverse effects on vessels and heart structures, and exposed to infection, malfunction and recalls.^[5]^ Long-term lead failure rates of up to 20% have been reported over a 10-year period.^[6]^ To address this issue, an entirely subcutaneous ICD (S-ICD) system has consequently been developed to reduce or even eliminate lead-related complications associated with trans-venous ICDs (TV-ICDs).

The clinical requirement to avoid venous access issues, endovascular mechanical stress producing lead malfunction, and extraction associated risks, as well as the apparent benefits, ultimately prompted more formal attempts to develop an entirely S-ICD.^[7]^ Its unique design avoids many drawbacks of TV-ICDs. The novel device, developed and tested over the past decade, has clinical evidence of efficacy and safety in detecting and terminating ventricular arrhythmias.^[8]^

Studies found that the S-ICD offers advantages in many aspects compared to a traditional trans-venous system, including lower complication rates and increased clinical benefit.^[6,9–13]^ Nevertheless, many of these reports came from small sample size or single-center trials, with inconsistent results. To overcome this paucity in the current literature, Basu-Ray et al made a meta-analysis of 5 case–control studies tried to summarize clinical outcomes between 2 groups, they found that S-ICD could reduce lead-related complications but was similar to TV-ICD with regard to nonlead related complications. However, This analysis was not comprehensive enough because it did not include outcomes as total complications, rate of ICD shocks, quality of life (QoL), procedure characteristics, length of stay, and so on, at the same time, some new studies were published recently, which prompted us to complete this analysis and make a more comprehensive assessment of S-ICD.

## 2 Methods

The Preferred Reporting Items for Systematic reviews and Meta-Analyses Amendment to the Quality of Reporting of Meta-Analyses Statement and Recommendations from the Cochrane Collaboration and Meta-analysis of Observational Studies in Epidemiology were followed during the development of the present systematic review.^[14,15]^

### 2.1 Data sources and search strategy

Relevant articles were searched in the Medline, EmBase, Cochrane Library, PubMed, and Elsevier's ScienceDirect databases. Reports published in non-English languages were excluded from the search. The terms “Sudden Cardiac Death” (all fields) OR “SCD” (all fields) OR “ventricular tachyarrhythmia” AND “implantable cardioverter defibrillator” (all fields) OR “ICD” (all fields) OR “transvenous implantable cardioverter defibrillator” OR “Transvenous ICDs” OR “TV-ICDs” AND “subcutaneous implantable cardioverter defibrillator” (all fields) OR “subcutaneous ICD” OR “S-ICD” were used as keywords. The literature search was updated in May 2018.

### 2.2 Inclusion and exclusion criteria

Two reviewers (M-JL and C-LJ) screened and identified studies that met the following inclusion criteria:

(1) patients received treatment with the implantable cardioverter defibrillator for SCD prevention;
(2) comparison between TV-ICD and S-ICD;
(3) sample size ≥20; and
(4) assessment of complications, infection rate, ICD therapy (appropriate and inappropriate therapies), and death rates.

Exclusion criteria were:

(1) inclusion of only patients with TV-ICD or S-ICD;
(2) equivocal study design or group allocation;
(3) conference abstracts, case reports, case series, editorials, and review articles.

### 2.3 Quality assessment and data extraction

Study quality was evaluated by an investigator (C-LJ) using the Newcastle–Ottawa Quality Assessment Scale for observational studies and Delphi consensus criteria for RCTs. Two independent investigators (M-JL and C-FC) abstracted the following data on prespecified forms: author's name, year of publication, country of study, number of enrolled patients, mean age, sex, body mass index, left ventricular ejection fraction, creatinine, follow-up duration, primary reason for preventive ICD, and study design. Data extraction was conducted by mutual agreement, and all potential disagreements were solved by consensus.

### 2.4 Assessment of heterogeneity reported bias and statistical analysis

A meta-analysis of the summary statistics from individual trials was performed. Statistical analysis was completed by an independent statistician (C-FC). Differences in dichotomous variables and outcome endpoints were reported as odds ratio (OR) or risk ratio (RR) with 95% confidence intervals (CIs). Continuous variables were analyzed using weighted mean differences or standard mean differences (SMD). Fixed-and random-effects models used weighting based on inverse variance calculated according to DerSimonian and Laird.^[16]^ Between-study heterogeneity was reflected by *I*^2^ > 50%, with a *P* < .05 deemed statistically significant. When no significant statistical heterogeneity was identified, the fixed effects model was preferentially used as the summary measure.

In case of statistical heterogeneity, sensitivity analyses were performed to assess the contribution of each study to the pooled estimate by excluding individual trials 1 at a time and recalculating the pooled RR estimate for the remaining studies.^[17]^ When pooled analysis still yielded significant heterogeneity, the random-effects model was used. Statistical analysis was performed with the Review Manager 5.3 software (The Nordic Cochrane Center, The Cochrane Collaboration, 2014, Copenhagen, Denmark).

### 2.5 Ethics

Ethical committee or medical institutional board approval was not required for systematic reviews and meta-analyses.

## 3 Results

### 3.1 Study selection

**Figure 1:**
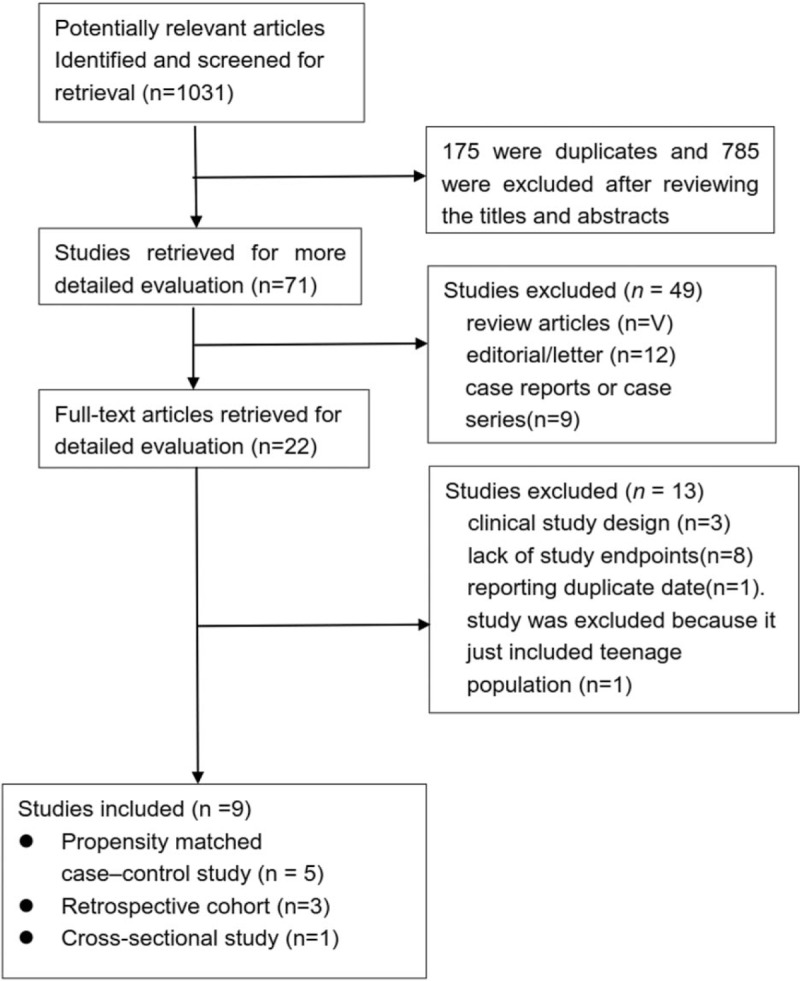
Flow diagram of studies selection for the exclusion/inclusion in this meta-analysis.

The selection procedure for the included clinical trials is shown in Figure 1. Initially, 1031 potentially relevant manuscripts were identified, of which 175 were duplicates; 785 were excluded after reviewing titles and abstracts. Of the 71 articles retained for further examination, 28 review articles, 12 editorials/letters, and 9 case reports or case series were excluded. Upon full-text assessment of the remaining 22 studies, 13 were excluded due to the following reasons: clinical study design (3); lack of study endpoints (8); only teenage population included (1),^[18]^ patients included coming from 2 studies (1).^[19]^ Finally, 9 clinical trials were included for analysis.

### 3.2 Study characteristics

The characteristics, indications and concomitant diseases of the 9 trials are summarized in Table 1 and Table 2. A total of 7361 patients were enrolled in these trials, including 2605 and 4756 in the S-ICD and TV-ICD groups, respectively.^[6,9–13,20–22]^ Mean ages of the study participants ranged from 35 ± 13 to 56.30 ± 12.71 years, and follow-up duration was 6 to 60 months. The percentage of men was 54.9% to 75%. Most patients received ICD therapy for primary prevention (50%–81%); in all trials, consecutive patients receiving treatment with TV-ICD were compared with age and sex-matched counterparts treated with the S-ICD. The grouping results ensured the feasibility of this meta-analysis.

**Table 1:**
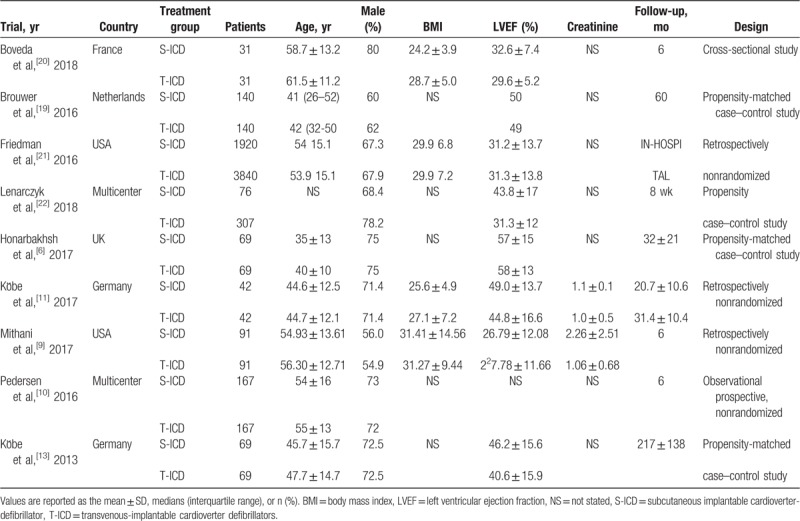
General characteristics of the included studies.

**Table 2:**
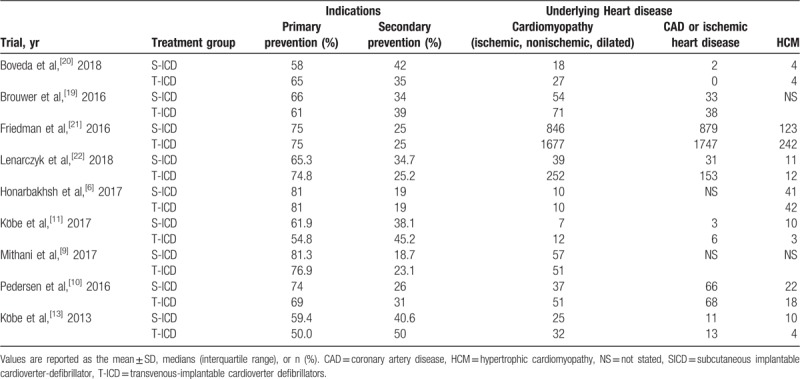
Indications and concomitant diseases of the included studies.

### 3.3 Complications

**Figure 2:**
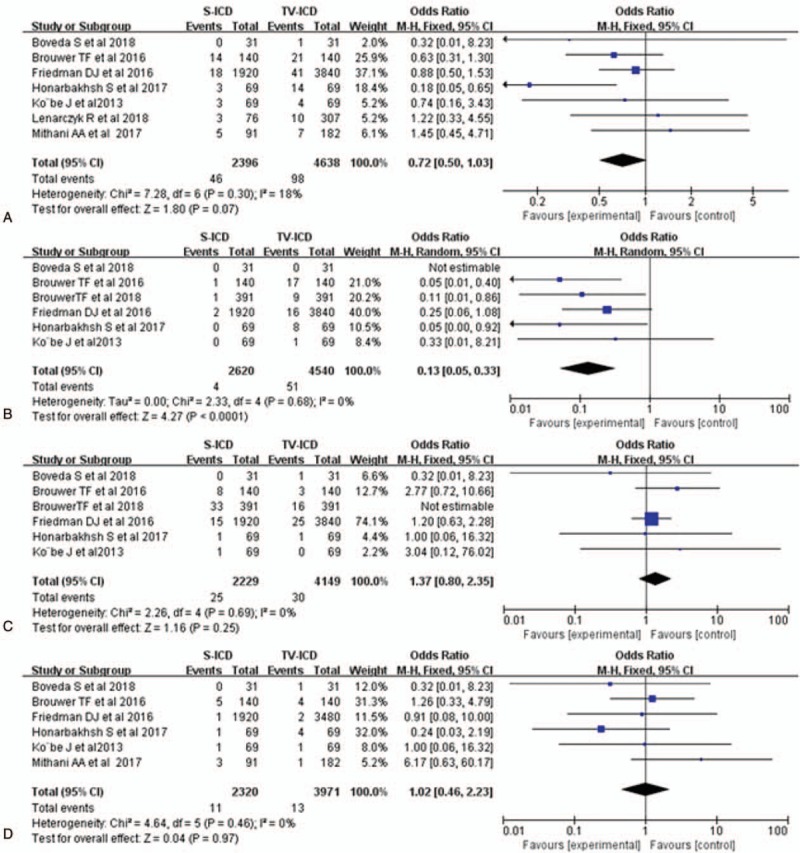
Forest plots of complications for S-ICD versus TV-ICD; Total complications (A); Lead related complications (B); Nonlead related complications (C); Infections (D). S-ICD = subcutaneous implantable cardioverter-defibrillators, TV-ICD = trans-venous implantable cardioverter-defibrillators.

Among the 2605 patients who received the S-ICD, 46 complications were found, versus 98 among the 4756 cases of the TV-ICD group. They included lead-related (migration, fracture, failure, and infection) and nonlead-related (pocket infection, delayed wound healing, wound discomfort, hematoma, device malfunction, and premature battery depletion) complications. Compared with TV-ICD treatment, pooled analysis of the included trials demonstrated that SICD had lower total complications (OR = 0.72; 95% CI, 0.50–1.03; *I*^2^ = 18% Fig. 2A), but the difference was not statistical. There was significant difference between 2 groups for lead-related complications (OR = 0.13; 95% CI, 0.05–0.33; *I*^2^ = 0% Fig. 2B). However, there was no significant difference in non-lead-related complications between the 2 groups (OR = 1.37; 95% CI 0.80–2.35, *I*^*2*^ = 0%, Fig. 2C). Device-related infections occurred in both groups, and infection rates were similar between the S-ICD and TV-ICD groups (OR = 1.02; 95% CI 0.46–2.23, *I*^2^ = 0%, Fig. 2D).

### 3.4 ICD shocks

**Figure 3:**
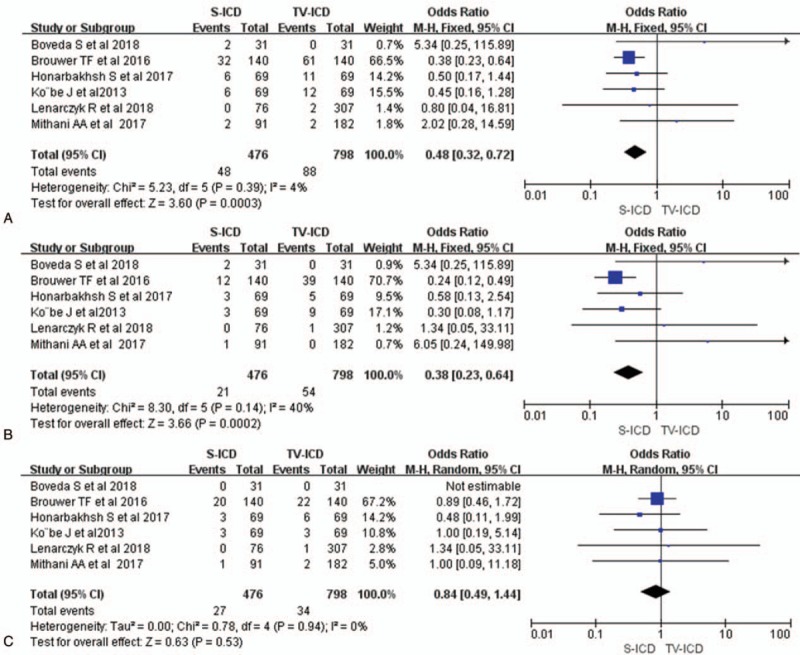
Forest plots of ICD therapy for S-ICD versus TV-ICD; ICD shocks (A); appropriate shocks (B); Inappropriate shocks (C). ICD = implantable cardioverter-defibrillators, S-ICD = subcutaneous implantable cardioverter-defibrillators, TV-ICD = trans-venous implantable cardioverter-defibrillators.

ICD shocks included appropriate and in-appropriate types. During follow-up, ICD shocks were significantly less in the S-ICD group compared with the TV-ICD group (OR = 0.48; 95% CI 0.32–0.72, *I*^2^ = 4%, Fig. 3A). For appropriate therapy, a total of 21 patients received appropriate shocks in the S-ICD group versus 54 in the TV-ICD group, indicating a significant difference (OR = 0.38; 95% CI 0.23–0.64, *I*^2^ = 40%, Fig. 3B). Moreover, the risks of inappropriate shocks were comparable between the 2 groups (OR = 0.84; 95% CI 0.49–1.44, *I*^2^ = 0%, Fig. 3C).

### 3.5 Mortality

**Figure 4:**
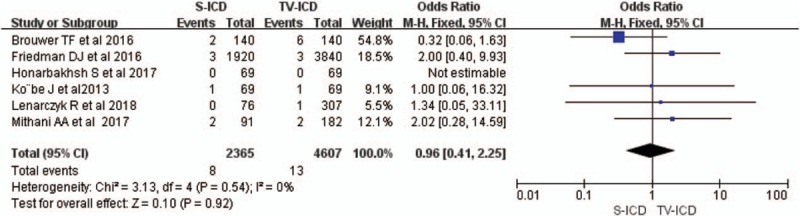
Forest plots of death for S-ICD versus TV-ICD. S-ICD = subcutaneous implantable cardioverter-defibrillators, TV-ICD = trans-venous implantable cardioverter-defibrillators.

Six trials in this meta-analysis reported mortality data, and no significant difference was observed between the S-ICD and TV-ICD groups (OR = 0.96; 95% CI 0.41–2.25, *I*^2^ = 0%, Fig. 4).

### 3.6 In-hospital outcomes

**Figure 5:**
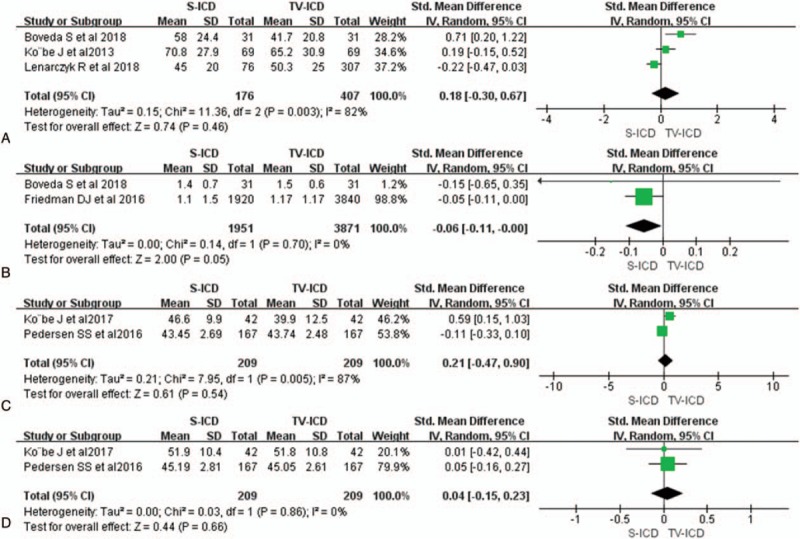
Forest plots of QoL and in-hospital outcomes for S-ICD versus TV-ICD; Procedure duration (A); Length of stay after implantation (B); Physical QoL (C); Mental QoL (D). QoL = quality of life, S-ICD = subcutaneous implantable cardioverter-defibrillators, TV-ICD = trans-venous implantable cardioverter-defibrillators.

Procedure durations were not significantly different between the S-ICD and TV-ICD groups (SMD = 0.18; 95% CI −0.03 to 0.67, *I*^2^ = 84%, Fig. 5A). In addition, compared with the TV-ICD group, patients who received the S-ICD tend to have shorter length of hospital stay after implantation, but the difference was not significant (SMD = −0.06; 95% CI −0.11 to 0.00, *I*^2^ = 0%, Fig. 5B).

### 3.7 Quality of life

Physical- and mental- well-being scores were used to evaluate the QoL. In this analysis, mental and physical QoL indicators were comparable in both groups (physical QoL: SMD = 0.21; 95% CI −0.47 to 0.90, *I*^2^ = 87%, *P* = .54, Fig. 5C; mental QoL: SMD = 0.04; 95% CI −0.16 to 0.27, *I*^2^ = 0%, *P* = .66, Fig. 5D).

## 4 Discussion

The present study may represent the most comprehensive meta-analysis so far comparing S-ICD and TV-ICD for efficacy, safety and in-hospital outcomes in the prevention of SCD. The principal findings were as follows:

(1) S-ICD treatment could significantly reduce lead-related complications, compared with the TV-ICD group;
(2) statistically significant ICD shock reduction was obtained in the S-ICD group compared with the TV-ICD group by pool analysis of the included studies;
(3) S-ICD has tendency to shorten length of hospital stay after implantation and reduce total complications, although the difference is no significant;
(4) for ICD implantation, the procedure duration, QoL, death and infection rates, and inappropriate shocks were comparable between the S-ICD and TV-ICD groups.

Over the past decade, there were 2 large prospective studies (IDE [S-ICD system IDE Clinical Investigation] and EFFORTLESS [Boston Scientific Post Market-S-ICD Registry]) have been conducted to evaluate the safety and efficacy of the S-ICD in large diverse populations.^[23,24]^ A pool analysis of these studies included 889 patients followed for an average of 1.8 years showed safety and efficacy of the S-ICD in patients with primary and secondary indications.^[25]^ Boersma et al published the midterm outcomes of EFFORTLESS study (nearly 1000 patients followed for an average of 3.1 years [3053 patient-years]), the outcome showed that the S-ICD fulfills predefined endpoints for safety and efficacy. Midterm outcome demonstrated rates on complications, inappropriate shocks, and conversion efficacy were similar to rates observed in TV-ICD studies.^[26]^

Due to its design, the S-ICD offers inherent advantages of eliminating the need for intravenous and intracardiac leads, as well as their associated risks and shortcomings.^[27]^ Many of these advantageous S-ICD design characteristics can also represent limitations. Its major disadvantage is the inability to deliver cardiac pacing and anti-tachycardia pacing (ATP) to terminate ventricular tachycardia (VT).^[28]^ Besides the limited pacing options, the system is larger (69.9 cm^3^) than conventional ICD generators, with a potential impact on the QoL and portability, especially in adolescents.^[29]^ Furthermore, because of the subcutaneous placement of the shocking electrode, the energy required for successful defibrillation is higher than that of TV-ICDs; the larger energy requirement for subcutaneous defibrillation may lead to increased defibrillation pain and shortened battery life, impacting the cost-effectiveness of this device.^[30]^ The many shortcomings and advantages of the S-ICD precisely prompted us to perform this analysis, aiming to achieve a more objective assessment of S-ICD.

### 4.1 Safety

There was a significant increase in lead-related complications in the TV-ICD group compared with the S-ICD group. The TV-ICD requires lead insertion into the central venous and placement in the ventricle; therefore, multiple complications can occur, including vascular obstruction, thrombosis, infection, and cardiac perforation, sometimes with catastrophic consequences.^[28]^ In addition, ICD lead performance is a serious concern over time. The potential for serious complications with transvenous-lead is substantial, and lead failure is estimated at 0.58%/yr and up to 20% at 10 years.^[4]^ Lead extraction may be necessary when lead failure occurs; this procedure is highly challenging with high complications rates of about 1% and a mortality risk of 0.3%, even in experienced centers.^[31]^ The S-ICD provides a defibrillation system without lead in or on the heart, thereby eliminating several important complications associated with transvenous leads. In the analysis, we found that the S-ICD could significant reduce the risk of lead-related complications by 87%.

Although the S-ICD system is larger than the TV-ICD, it did not increase the risk of nonlead-complications. Moreover, device-related infection showed no statistical difference between the 2 groups. Therefore, the S-ICD does not increase nonlead-related complications and infection risk compared with TV-ICD.

The TV-ICD is effective in improving survival in patients at increased risk of SCD, and many studies also found that the S-ICD could successfully stop ventricular tachycardia. In this analysis, the mortality rate was low, and both devices appeared to perform equally with respect to reducing mortality.

### 4.2 Efficacy

ICDs effectively stop ventricular tachycardia, but recurrent ICD shocks may impair the QoL, and are associated with increased risk of death, heart failure, and hospitalization; patients with both appropriate or inappropriate shocks more often require suppressive therapy compared with those who have received no shocks.^[32,33]^ In this analysis, the S-ICD could significantly reduce the rate of appropriate shocks, which may be due to the ability of TV-ICDs to deliver ATP instantly after VT detection, whereas the S-ICD has a longer charging time, which allows non-sustained VTs to terminate.^[12]^ Previous reports indicated that inappropriate shocks affect 4.3% (range 0%–15%, 2.9% per person-years of follow-up) of patients receiving an S-ICD, a frequency similar to the observed rate reported in previous TV-ICD trials.^[34]^ As shown above, comparable rates of inappropriate shocks were found between the 2 groups, with different mechanisms. The majority of inappropriate shocks in TV-ICDs are supra-ventricular tachycardia while those associated with S-ICDs are over-sensing T waves (up to 80%) or myopotential signals.^[23,25]^ Inappropriate shocks from the S-ICD caused by over-sensing T waves can be managed and prevented by subsequent introduction of dual zone programming and reprogramming of the sensing vector. So exercise testing shortly after implantation may be considered in patients at high risk of T wave over-sensing.^[19,26,35,36]^ Gold et al performed a head-to-head comparison of arrhythmia discrimination performance between the S-ICD and TV-ICD, and found that appropriate detection rates of ventricular tachyarrhythmia for S-ICD and TV devices in single- and dual-zone configurations are 100% and >99%, respectively. Specificity for supra-ventricular arrhythmia was significantly improved for the S-ICD system compared with 2 of 3 TV systems, as well as composite TV devices.^[27]^

Moreover, because of the subcutaneous placement of the shocking electrode, the energy required for successful defibrillation is higher with the S-ICD than the TV-ICD. Larger volume and higher energy shocking may affect more the patient's QoL. Kobe et al performed a study aimed at comparing the QoL and posttraumatic stress disorders (PTSD) in patients after treatment with S-ICD and TV-ICD, respectively. They found that constant or even improved physical well-being of patients with the S-ICD and PTSD was comparable between the 2 groups. Pedersen et al evaluated the QoL of patients with an S-ICD against an unrelated cohort with a TV-ICD system in a 6-month follow-up. They found that both groups experienced significant improvements in physical and mental QoL from the time of implant.

### 4.3 In-hospital outcomes

Some argue that the S-ICD is technically more challenging, and the systematic defibrillation threshold testing may lead to somewhat longer procedure duration.^[24]^ The clinical outcomes show inconsistency. In the EFFORTLESS S-ICD Registry the S-ICD was performed in 69 ± 27 minutes, comparable to studies by Boveda et al and Köbe et al; however, Lenarczyk et al reported shorter procedure time (45 ± 20 minutes).^[13,20,22,26]^ A pooled analysis of 3 trials included in this analysis revealed similar procedure durations between the 2 groups. On the other hand, the TV-ICD may prolong the length of hospital stay after implantation, likely because of prolonged pain management and peri-procedural complications, among others.

This meta-analysis had several limitations. First, publication bias could not be completely excluded, as with any literature search of databases, and inclusion of only published data contributed to bias. Second, a potential risk of pooling data from different studies was mixing patients with different clinical characteristics. Third, more well-designed and large-scale RCTs are required to confirm the reported findings. Fourth, shock efficacy is one of the most important endpoints for comparing the S-ICD and TV-ICD systems, but we did not perform further analysis due to limited sample size. Finally, most studies involved Caucasians from Western communities, which limits the generalizability of the current findings.

The S-ICD represents an important advancement in the ICD technology for the last 15 years. We hope next S-ICD generation devices may solve current limitations such as pacing capability and remote monitoring capability, downsizing the generator and improving the battery technology in the future.

## 5 Conclusions

The present systematic review and meta-analysis demonstrated that S-ICD has some advantages over TV-ICD, including reducing lead-related complications and ICD shocks. In addition, S-ICD has a tendency to shorten the hospitalization and reduce total complications, although the difference is no significant. Moreover similar death rate, infection rate, QoL, and procedure time were found between 2 groups. Therefore, with appropriate patient selection, the S-ICD may be emerged as an alternative to TV-ICD for SCD prevention.

## Author contributions

Dr Chao-Feng Chen and Dr Mei-Jun Liu: contributed to evaluate and write the manuscript. Chao-Lun Jin: contributed to statistical analysis and helped gather references for the manuscript. Dr Yi-Zhou Xu: contributed to conceive and design the experiment and revise the manuscript.

**Conceptualization:** Mei-Jun Liu, Yi-Zhou Xu.

**Data curation:** Mei-Jun Liu.

**Methodology:** Chao-Feng Chen, Mei-Jun Liu, Chao-Lun Jin.

**Resources:** Mei-Jun Liu, Chao-Lun Jin.

**Software:** Chao-Feng Chen, Chao-Lun Jin.

**Supervision:** Yi-Zhou Xu.

**Validation:** Yi-Zhou Xu.

**Visualization:** Yi-Zhou Xu.

**Writing – original draft:** Chao-Feng Chen.

**Writing – review and editing:** Chao-Feng Chen, Yi-Zhou Xu.
